# Behavioral and Neural Arguments of Motivational Influence on Decision Making During Uncertainty

**DOI:** 10.3389/fnins.2020.00583

**Published:** 2020-06-05

**Authors:** Julie Giustiniani, Magali Nicolier, Juliana Teti Mayer, Thibault Chabin, Caroline Masse, Nathan Galmès, Lionel Pazart, Benoit Trojak, Djamila Bennabi, Pierre Vandel, Emmanuel Haffen, Damien Gabriel

**Affiliations:** ^1^Department of Clinical Psychiatry, University Hospital of Besançon, Besançon, France; ^2^EA 481, Laboratory of Neurosciences, University of Burgundy Franche-Comté, Besançon, France; ^3^Clinical Investigation Centre, University Hospital of Besançon, Besançon, France; ^4^Neuroimaging and neurostimulation department Neuraxess, University of Burgundy Franche-Comté, Besançon, France; ^5^Fondation FondaMental, Hôpital Albert Chenevier, Créteil, France; ^6^Department of Psychiatry and Addictology, University Hospital of Dijon, Dijon, France; ^7^EA 4452, LPPM, University of Burgundy Franche-Comté, Dijon, France

**Keywords:** decision-making, uncertainty, motivation, IGT, EEfRT, P300, effort

## Abstract

The scientific world is increasingly interested in motivation, primarily due to the suspected impact on decision-making abilities, particularly in uncertain conditions. To explore this plausible relationship, 28 healthy participants were included in the study and performed decision-making and motivational tasks while their neural activity was recorded. All participants performed the Iowa Gambling Task (IGT) and were split into two groups based on their score, one favorable group with 14 participants who performed advantageously and one undecided group with 14 participants who failed to develop the correct strategy on the IGT. In addition, all participants performed the Effort Expenditure for Reward Task (EEfRT), which defines the motivational level of each participant by the effort that participants agree to do in function of reward magnitudes and probabilities to receive these reward (10, 50, and 90%). The completion of both tasks allowed for the exploration of the relationship between the motivational level and decision-making abilities. The EEfRT was adapted to electroencephalography (EEG) recordings to explore how motivation could influence reward experience. Behavioral results showed no difference in EEfRT performances on the whole task between the two groups’ performances on the IGT. However, there was a negative correlation between the difficulty to develop an optimal strategy on the IGT and the percentage of difficult choices at the 90% condition on the EEfRT. Each probability condition has been previously associated to different motivational and emotional states, with the 90% condition associated to the reward sensitivity. This behavioral result leads to the hypothesis that reward sensitivity may induce an inability to develop an optimal strategy on the IGT. Group analysis demonstrated that only the undecided group showed a P300 during the processing of the outcome, whereas the favorable group showed a blunted P300. Similarly, there was a negative correlation between the P300 amplitude and the ability to develop an optimal strategy on the IGT. In conclusion, behavioral and neuronal data provides evidence that the propensity to focus only on the immediate outcomes is related to the development of an inefficient strategy on the IGT, without influence of motivation.

## Introduction

The scientific world is increasingly interested in motivation, both as a function of the alteration in various neuropsychiatric disorders (Treadway et al., 2012, 2014) as well as through its influence on various cognitive processes, such as attention (Fan et al., 2002; Engelmann and Pessoa, 2007; Pessoa, 2009; Robinson et al., 2012; Oliveira et al., 2013), working memory (Taylor et al., 2004), long-term memory (Nielson and Bryant, 2005), and cognitive control (Chiew and Braver, 2014). It has been previously demonstrated that motivation plays an important role in the performance of various neurocognitive tests (Locke and Braver, 2008). While the Iowa Gambling Task (IGT) is a well-known laboratory task designed to assess decision-making ability during uncertain conditions and it has been used in multiple, various situations (Bechara et al., 1994, 1997), the influence of motivation on participant performances on the IGT has yet to be evaluated. The IGT was originally created to study decision-making impairments in patients with ventromedial prefrontal cortex damage, however, many IGT studies have shown large inter-individual variability regarding their performance in a healthy population. Several clinical reports have reported that, while a majority of healthy participants are able to develop an optimal strategy on the IGT, others do not acquire a preference for one deck over the others, indicative of a lack of learning. In fact, several studies have reported between 37 and 55% failure in healthy population in the IGT (Bechara et al., 2001; Bechara and Damasio, 2002; Bagneux et al., 2013; Mapelli et al., 2014; Giustiniani et al., 2015). The literature has suggested many factors that could account for this variability in IGT performance (e.g., low educational and intellectual levels), but cannot fully explain the variability. Motivation may be one element that explains the heterogeneity of the performance on the IGT. Motivation is identified indirectly (i.e., motivational levels can’t be controlled) as an important element in the decision-making process in uncertain conditions (Singh, 2013; Giustiniani et al., 2015), therefore, many studies use monetary reward to improve the involvement in performing the task (Killgore et al., 2006). Additionally, Voss et al. (2008) used a color discrimination task to demonstrate that results in ambiguous situations were more influenced by the motivational level. Furthermore, both motivation and decision-making under uncertainty seem to be altered in the same neuropsychiatric (Cella et al., 2010; Treadway et al., 2012, 2014; Kim et al., 2016) and addictive disorders (Brevers et al., 2016), lending support for a link between these two concepts. Moreover, in an electrophysiological study using event-related potentials (ERPs), a P300 was observed following a loss of money in participants able to develop a correct strategy at the IGT (Giustiniani et al., 2015). The P300 is one of the primarily studied ERPs, known to play an important role in reward processing (Sutton et al., 1965; Wu and Zhou, 2009) and in a large number of cognitive and affective processes (Polich, 2007). Furthermore, the P300 has also been linked with motivational processes (Nieuwenhuis et al., 2005) and its amplitude was described to be proportional to the motivational level (Yeung and Sanfey, 2004). Although the link between motivation and decision making is currently suspected and indirectly made, the evidence of a direct relationship between these two processes has yet to be made (Giustiniani et al., 2017).

The role of motivation is complex and, subsequently, difficult to study (Ryan and Deci, 2000), therefore, it should be clearly defined and evaluated. The concept of motivation can be defined in terms of goal-directed behaviors, such as efforts engaged in the actions conducted to obtain the expected results (Braver et al., 2014). Motivation occurs during the triggering of one activity, but also occurs while the activity continues (Schunk, 2000). Motivation could be defined in cognitive neuroscience as the neural representations of expected outcomes that predict decisions regarding effort investment (Braver et al., 2014). Among the various methods to evaluate motivation, the Effort-Expenditure for Reward Task (EEfRT) (Treadway et al., 2009) appears to be the most relevant task, because it translates the concept of motivation in a behavior in terms of effort to obtain a reward (van den Bos et al., 2006). The EEfRT was originally developed to evaluate motivational dysregulation in clinical populations and its use has been validated on several different populations, such as those with mood disorders, schizophrenia (Whitton et al., 2015), obesity (Mata et al., 2017), and in cannabis users (Lawn et al., 2016), demonstrating its wide acceptability. More precisely, the EEfRT is a multi-trial task in which participants are asked to choose between two options (one easy and one difficult) as a function of the magnitude of the monetary reward and the probability of receiving this reward (between 12, 50, and 88%) if the task is completed. Each option is associated to a button press effort. In difficult options the participant is asked to make a large number of button presses with its no-dominant hand whereas in the easy option less presses are requested, this time with the dominant hand (Treadway et al., 2009). It is important to understand that effort is explained in terms of various costs such as physical effort, uncertainty, and delays to receipt reward. In addition, each probability condition (12, 50, and 88%) could be associated with motivational and emotional states. Indeed, subjects with more motivation made significantly more hard choices than easy choices when the probability to receive the reward is low (12%), in order to receive a greater final gain (Wardle et al., 2011). While the middle probability (50%) condition appears to be sensitive to the lack of motivation in an anhedonic population (Treadway et al., 2009), the high probability condition (88%) seems sensitive to the anticipation of pleasure (Yang et al., 2014).

Behavioral measures of motivation do not account for the dynamic construction of the motivational process. In its neuroscientific definition, motivation is indeed strongly associated with the reward experience and more precisely with neural representations of expected outcomes that predict decisions regarding effort investment (Braver et al., 2014). Moreover, reward experience is a construct characterized by distinct processes, categorized as outcome processing, reward learning, and reward anticipation (Berridge et al., 2009; Berridge and Kringelbach, 2015). To study the dynamic aspect of motivation, the use of neuroimaging with high temporal resolution appears to be one method of interest. Using the high-resolution electroencephalography (HR-EEG), whose high temporal resolution brings in a dynamic view the different stages of the reward experience and whose spatial resolution gives the opportunity of localizing the neural structures involved in these processes (Nahum et al., 2011; Wahlen et al., 2011). In this context, we adapted the EEfRT to allow for the analysis of the ERP, thus providing the identification of various neurophysiological markers of motivation, such as the P300 and the stimulus preceding negativity (SPN). The SPN is a non-motor expectancy wave preceding a relevant stimulus, during which a non-motor response is required (Brunia and Damen, 1988). The SPN reflects reward anticipation, with a greater negativity when there is a possibility to receive desirable outcomes (Fuentemilla et al., 2013).

The aim of the current study was to investigate the relationship between motivation and decision-making under uncertain conditions. For that purpose, we selected 28 healthy participants, who performed versions of the IGT and the EEfRT adapted to study ERPs (Giustiniani et al., 2015, 2019). We hypothesized that the ability to develop an efficient strategy on the IGT could be explained by high motivational levels at the EEfRT. As a first step, the existence of a behavioral relationship between IGT and EEfRT performances was explored. In the next step, we measured ERPs resulting from the EEfRT in order to explore the influence of motivation during reward experience. We could therefore examine if the ERPs related to specific stages of the reward process, such as the SPN and the P300, were predictive of the IGT performances.

## Materials and Methods

### Population

Thirty-two healthy, right-handed subjects, all males (mean age: 25 ± 5.29) participated in the current study. No participants had any previous medical history of psychiatric disorders, substance abuse, alcohol abuse, neurological diseases, traumatic brain injury or stroke, nor did they take any medication. Prior to participating in the study, participants received information regarding the aims and procedures of the experiment and gave their written informed consent to participate. The influence of real money playing a significant role in motivation, subjects received information that the monetary payment would be proportional to the global gain obtained in the IGT and the EEfRT. Due to ethical considerations, regardless of their performance, all participants received the maximum amount of 75€ at the end of the experiment. All methods were performed in accordance with the relevant guidelines and regulations and all methods were approved by the Ethics Committee of Besançon University Hospital [authorized by the General Health Administration (ANSM 2016-A00870-51)].

### Experimental Tasks

All participants performed both the IGT and the EEfRT, in a randomized order.

#### Iowa Gambling Task

The task was an electronic version of the IGT, adapted for the study of ERPs and the analysis of brain activity sources. The aim of the task was to win as much money as possible by making successive selections between four decks.

The composition of decks, values, and schedules of reward/punishment were predetermined identically to the original form of the IGT (Bechara et al., 1994). While the back of each deck looked identical, they differed in composition. Decks A and B were the disadvantageous decks, they provided immediate reward, but in the long run yielded major economic losses. Decks C and D were the advantageous decks, they provided frequent small wins and smaller long term penalties, which resulted in long-term gain. The subjects were not informed of the number of trials they would be playing. To adapt the IGT to our French population, the money used to play was converted from US Dollars to Euros. At the beginning of the IGT, participants received a loan of 2,000€.

A few changes had to be made to adapt the IGT task to work with the EEG. First, to extend the electrophysiological recording from the hunch phase, no specific instructions were given to participants regarding the presence of advantageous or disadvantageous decks. In the absence of the instructions, the final performance usually worsened, therefore, the exploration phase was longer, and the optimal strategy was hardly found in the 100 trials. However, when more trials were allowed, many individuals performing poorly in the first 100 trials are able to achieve a good final performance. To that purpose, the number of trials was increased from 100 to 200. Each deck contained 200 cards. Second, the design of the trial process was modified to minimize ocular artifacts. For each trial, subjects were required to focus on a cross or a letter while making their selection by pressing a key. After the selection, a feedback of the deck chosen and the total credit amounts were displayed, followed by the amount of money involved in this trial. Then, a fixation point appeared to focus the eyes, followed by a green square if the money was won or a red square if the money was lost. Subjects received instructions to focus on the square and not to blink as long as they had not made their next selection. The choice to show a letter and not the amount of money and outcome simultaneously was made to avoid ocular movements induced by reading the amount. Before beginning the task, subjects were trained with a 5-trials short version of the game.

#### Effort Expenditure for Reward Task

The Effort Expenditure for Reward Task (EEfRT) was modified from the original version (Treadway et al., 2009) and adapted for ERP analysis. The goal of the EEfRT is to win as much money as possible by completing either easy or hard tasks. Each task is selected as a function of the amount of money that can be won if the task is completed as well as the probability of receiving the reward when the task is completed. This adaptation of the EEfRT was programmed in E-prime (Psychology Software Tools Inc., Sharpsburg, PA, United States). Both the probability, as well as the order of amounts, were randomized across participants. To ensure task comprehension, subjects received oral instructions and were provided with a series of task instructions, followed by a few practice trials prior to starting the experiment.

The experiment began with a calibration phase, consisting of determining the maximum number of button presses that participants were able to perform in seven seconds with the index finger of their right hand and in fourteen seconds with the auricular finger of the left hand, allowing for personalization of the difficulty of the EEfRT.

In the adaptation used in the current study, the number of trials was fixed to 120. To complete the easy task, participants were required to execute 70% of their maximum number of buttons presses obtained with the right index finger in the calibration phase within seven seconds. When the easy task was completed, participants were eligible to win 1€. For the hard task, participants were required to execute 90% of their maximum number of buttons presses obtained in the calibration phase with the auricular finger of the left hand within 14 s. The time assigned for the completion of the hard task was reduced compared to the original task of 21 s, in order to compensate for the increased number of trials, which increase the study time. When the hard task was completed, participants were eligible to win either 1.5€, 3€, 4.5€, or 6€ (instead of a range of $1.24–$4.30 in the original version). The values were adapted to our French population with European money. Probabilities to win the money when the task was completed have been changed to 10, 50, or 90% (instead of 12, 50, and 88% in the original version). These probabilities applied to both the hard and the easy tasks and were distributed in equal proportions across the experiment. Effort was evaluated by the proportion of choice High Reward / High Cost (HR/HC) or Low Reward / Low Cost (LR/LC) choices on the whole task as function of each probability condition.

### Group Assignment

According to their performance on the IGT, participants were separated into two equal groups, those able to develop a favorable strategy and those who were not.

The 200 trials were divided into 10 blocks of 20 trials and, for each block, the net score was calculated by subtracting the number of disadvantageous decks from the number of advantageous decks selected. In order to specifically examine the neural mechanisms underlying the elaboration of a successful long-term strategy on the current task, the net scores from the conceptual phase (i.e., from the last blocks at which the net score remained stable) were used to categorize participants.

The net score was considered to have remained stable when the overall performance was significantly different from a random choice of advantageous and disadvantageous selections. Bonferroni corrected *t*-tests were used to compare the evolution of the gambling performance from chance. From the 4th block on (i.e., 60th trial), participants’ net score was significantly different from zero. Per previous studies, subjects were then classified, post hoc, into two groups differing in net score in blocks 4–10, favorable if the net score was higher than 10, unfavorable if the net score was less than –10 and undecided if the net score range was between 10 and – 10 (Bechara et al., 1994, 2000a; Bechara and Damasio, 2002; Giustiniani et al., 2015, 2019). In the pool of 32 participants, fourteen subjects were found to develop a correct strategy and were assigned to the favorable group (mean age: 25.4 ±/ 5.9). Therefore, fourteen participants were randomly selected on the 18 remaining participants unable to develop a correct strategy and were assigned to the undecided group (mean age = 23.8 ± 4.07). No significant differences were observed between each group concerning years of study (*p* = 0.310), marital status (single, couple, divorced) (*p* = 0.159), and the professional status (student, employment, unemployment) (*p* = 0.275). The term undecided was used to highlight that subjects from this group were unable to move toward a positive or negative strategy. These participants favored neither advantageous nor disadvantageous decks (Giustiniani et al., 2015, 2019). Table 1 shows the net scores for both groups.

**TABLE 1:** EEfRT and IGT scores in the favorable and undecided groups.

	**EEfRT**										**IGT**
	
	**Probability (% difficult choices)**			**Amount (% difficult choices)**				**Amount of money**	**Button presses**		**net score (blocks 4–10)**
	**10%**	**50%**	**90%**	**1.5**	**3**	**4.5**	**6**		**Hard task**	**Easy task**	
Favorable group	8%	41%	61%	2%	35%	50%	61%	135.32	82.07	40.14	16.87
Undecided group	8%	48%	75%	13%	42%	57%	61%	150.79	86.14	41.36	1.35

### EEG Recording

EEG signals were recorded using a 256 channel Geodesic Sensor Net (Electrical Geodesics Inc., EGI, Eugene, OR, United States) during both the IGT and EEfRT. All channels were referenced to the vertex (Cz) and collected with a high impedance amplifier (Net Amp 300 amplifier, Electrical Geodesics) using Net Station 4.5 software (Electrical Geodesics). Data were continuously recorded using a high-pass filter at 1 Hz with a sampling rate at 1000 Hz. For both the IGT and the EEfRT, subjects were instructed to limit body movements, eye blinks, and muscular contractions during task selection and reward feedback.

### Data Analysis

#### Behavorial Data Analysis

In addition to the IGT net score, which was calculated to separate the participants in two groups, several data were extracted from the EEfRT. Two categories of data were analyzed. First, overall motivation as the number of button presses (measured during the calibration phase) and the number of completed trials for the easy and hard tasks was analyzed. Second, parameters relative to the strategy developed at the EEfRT were analyzed as the number of difficult choices of the participant as a function of the different amounts of money and the probabilities of winning the money. Proportion’s calculation of choices for the HR/HC or LR/LC was conducted on the whole performance task and in the second step on each probability. We have seen previously that each probability translated a motivational state (Treadway et al., 2009; Wardle et al., 2011; Yang et al., 2014).

#### EEG Data Analysis

EEG data analysis was performed using Cartool Software 3.55^1^. Raw EEG data were re-referenced offline to a common average reference.

Analyses were conducted for the EEfRT on two intervals around the reward screen. The main temporal interval of interest was following the reward. Epochs of 700 ms (100 ms prior to reward feedback – 600 ms following reward feedback) were extracted from the raw data and analyzed, with a baseline correction applied prior to the feedback through the onset of the feedback (100 ms – 0 ms). The P300 was defined as the mean voltage between 290 and 410 ms, based on grand averages of ERPs for the rewarded and not rewarded conditions. An additional analysis of the FRN, an ERP reflecting the early processing of the outcome, being defined as the mean voltage from 240 to 290 ms, was also conducted. The temporal interval preceding the reward, computed for easy and hard tasks, was also analyzed and related to the SPN. Epochs of 600 ms (500 ms prior to the outcome – 200 ms after) were extracted from the raw data, with a baseline correction of 100 ms applied prior to the participant selection of an easy or hard task. The SPN was defined as the mean voltage within 200 ms prior to the reward feedback. Due to a large number of artifacts, the SPN of three subjects from the advantageous group were removed from subsequent analyses.

For the IGT, the main interval of interest came following the reward screen. Epochs of 700 ms (100 ms prior to reward feedback – 600 ms following reward feedback) were extracted from the raw data and analyzed, with a baseline correction applied prior to feedback on the onset of the feedback (100–0 ms). The P300 was defined as the mean voltage between 290–440 ms, based on grand averages of ERPs for win and loss conditions.

For all ERPs, a band pass filter was applied between 1–30 Hz and a notch filter was applied at 50 Hz to remove environmental artifacts. A semi-automatic artifact rejection method was used, with a fixed criterion of ±100 μV. Remaining epochs were visually inspected, manually removing any containing blinks, eye movements, or other sources of transient noise from the analysis. Electrodes with an aberrant signal (e.g., excessive noise due to malfunctioning or a bad signal during data collection) were interpolated using a 3-dimensional spline algorithm (average: 4.67% interpolated electrodes). Per previous literature on feedback processing, six central electrodes (Fpz, Fz, FCz, Cz, CPz, Pz) were chosen for the current analysis.

To visualize the brain regions accounting for the different ERPs, source localization was applied using a distributed linear inverse solution based on a Local AUto-Regressive Average (LAURA) model, comprising a solution space of 3005 nodes. Current distribution was calculated within the gray matter of the average brain, provided by the Montreal Neurological Institute (MNI).

#### Statistics

The overall motivation measured for the EEfRT (i.e., the number of button presses and the number of completed trials) was compared for the two groups of participants (advantageous/undecided) by using paired *t*-tests. The strategies developed at the EEfRT were analyzed by using partially repeated-measures analysis of variance (ANOVA) with three factors, namely group (advantageous/undecided), sum (1.5 to 6 euros), and probability (10, 50, and 90%). The ERPs measured for the EEfRT (P300, FRN) and for the IGT (P300) were also analyzed by using partially repeated-measures analysis of variance (ANOVA) with three factors, namely group (advantageous/undecided), electrodes (FPz, Fz, FCz, Cz, CPz, Pz) and outcome (win or loss). For the SPN, a partially repeated-measures analysis of variance (ANOVA) with three factors was used, namely group (advantageous/undecided), electrodes (FPz, Fz, FCz, Cz, CPz, Pz), and task (easy or hard). In all of these analyses, the threshold of significance was set to 5% and post hoc analyses were performed using a Bonferroni correction.

To evaluate whether the IGT net score and EEfRT parameters (behavior and ERP) were related, nonparametric Spearman rank-order correlations were used. Behavioral EEfRT parameters were the proportion of choices for each probability condition, as well as the total amount of money won by participants. Neural EEfRT parameters were mainly the amplitude of the 6 different electrodes during the P300, but also during the SPN (in a more exploratory approach). Similar correlations were used to compare P300 responses on the IGT and the IGT net score. To consider multiple comparisons, the threshold of significance was set to 1%. We performed the analyses using Statistica 11.0 for Windows (StatSoft, Inc., Tulsa, OK, United States).

## Results

### Behavior at the EEfRT

Table 1 shows behavioral performances at the IGT for the favorable and undecided groups.

First, we wanted to evaluate whether the ability to develop a strategy for the IGT was related to the behavioral performance on the EEfRT. There were no difference in overall motivation, demonstrated by no differences between both groups in the number of button presses neither for the difficult task [*t*(26) = −1.00, *p* = 0.39] nor for the easy task [*t*(26) = −0.58, *p* = 0.18]. Similarly, there was no difference in completing the difficult task [*t*(26) = 0.04, *p* = 0.97] or the easy task [*t*(26) = 0.92, *p* = 0.36] for each group.

A 2 way partially repeated measures ANOVA with the factors group (favorable/undecided) and sum (1.5–6€) revealed that the decision-making differences on the IGT did not influence the strategy on the EEfRT (ANOVA partially repeated [*F*(1,26) = 1.94, *p* = 0.18)]. Similarly, a two way partially repeated measures ANOVA with the factors group (favorable/undecided) and probability (10, 50, and 90%) showed that decision-making differences on the IGT did not influence choices based on the probability of gain at the EEfRT [*F*(1,26) = 2.02, *p* = 0.16].

**FIGURE 1:**
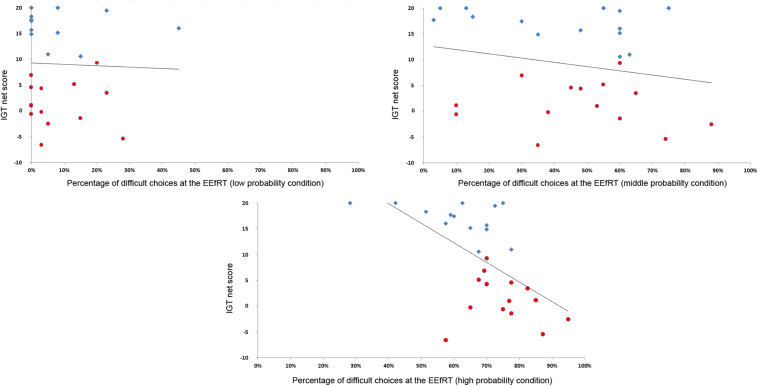
Correlation between the netscore at the IGT and the percentage of difficult choices. The more participants developed a correct strategy at the IGT, the less they selected difficult choices at a probability of 90%. For visualization purpose, participants of the advantageous group are represented in blue diamonds and participants of the undecisive group in red circles.

However, when looking at the relationship between the IGT and the EEfRT at the individual level, there was a strong correlation between IGT performance and the percentage of difficult choices at a probability of gain of 90% (*r*^2^ = −0.59, *p* < 0.001) (Figure 1).

### Event-Related Potentials at the EEfRT

**FIGURE 2:**
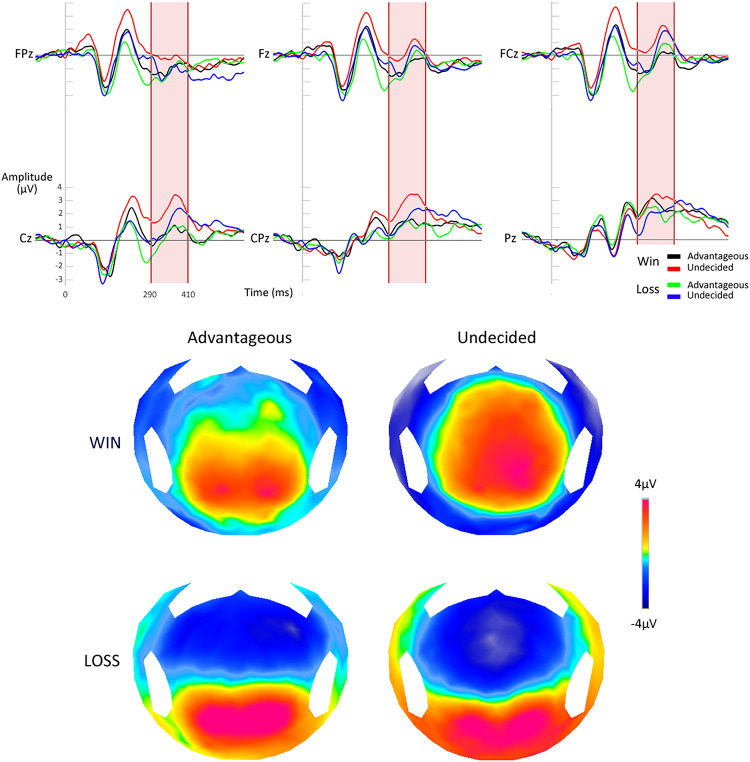
Electrophysiological responses after the processing of a reward at the EEfRT. Top: ERPs on the six electrodes of interest. Down: topographic maps for the four conditions.

The analysis of the P300 revealed that the amplitude of the evoked potential related to the processing of the reward or the absence of the reward on the EEfRT differed significantly in function of the ability to develop a correct strategy or not at the IGT [*F*(1,26) = 4.83, *p* < 0.05]. More precisely, the mean P300 amplitude was larger in the undecided group after a gain compared to an absence of gain (*p* < 0.01). This difference was not present in the favorable group (*p* = 1) (Figure 2). No such effect was seen when analyzing the early processing of the outcome, with the FRN [*F*(1,26) = 0.09, *p* = 0.77].

**FIGURE 3:**
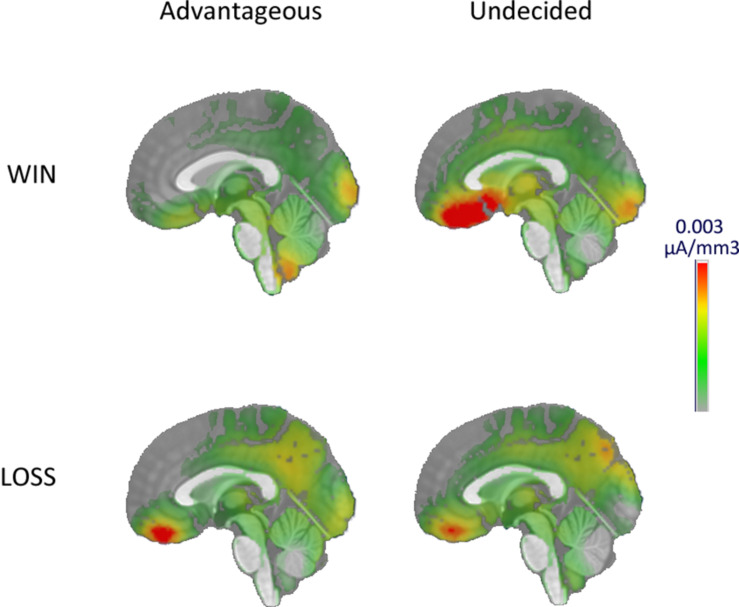
Source imaging of the P300 at the EEfRT.

To visualize from which neural structure the differences of P300 topography originated, source localization was performed on the P300 responses. A larger activity in the ventromedial prefrontal cortex was observed in the undecided group after a win (Figure 3).

**FIGURE 4:**
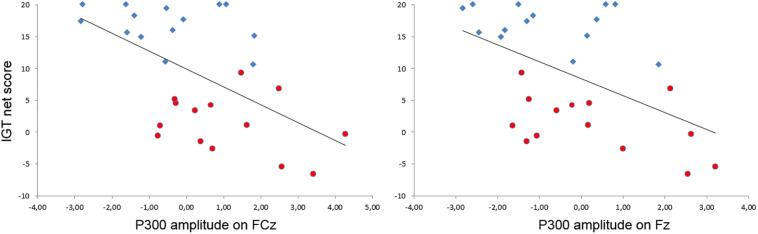
Relationship between the amplitude of the P300 after a gain at the EEfRT and the development of a strategy at the IGT. A significant inverse correlation was observed on electrodes FCz (left) and Fz (right). For visualization purpose, participants of the advantageous group are represented in blue diamonds and participants of the undecisive group in red circles.

A relationship between the strategies developed on the IGT and the amplitude of the P300 on the EEfRT was also observed on most of the frontal electrodes (Figure 4). Indeed, the more subjects developed an undecided strategy on the IGT, the higher the amplitude of the P300 during a gain on the EEfRT on the Fz (*r* = −0.50, *p* < 0.01) and FCz (*r* = −0.55, *p* < 0.01) electrodes.

We also performed an exploratory analysis of the anticipation of the reward. The analysis of the SPN did not show any influence of the decision-making strategy on its amplitude, either when waiting for the result after a difficult task or after an easy task [*F*(1,23) = 0.14, *p* = 0.71].

### Event-Related Potentials at the IGT

In order to replicate previous results (Giustiniani et al., 2015), we examined whether IGT behavior also had an impact on the evoked potentials recorded during the IGT. The current results demonstrated that the amplitude of the P300 differed significantly as a function of the ability to develop a correct strategy or not on the IGT [*F*(1,26) = 4.45, *p* < 0.05], with a stronger amplitude for the undecided group. Similar to what was observed with the EEfRT at the individual level, a link between the amplitude of the P300 wave in the reward condition and the IGT score was observed, but on more posterior electrodes. The more subjects developed an undecided strategy on the IGT, the higher the amplitude of the P300 wave was during a gain on electrode Cz (*r* = −0.48, *p* = 0.01) and on neighboring electrodes at lower significance levels (FCz: *r* = −0.42, *p* < 0.05, and CPz: *r* = −0.42, *p* < 0.05).

## Discussion

In the current study, we evaluated the relationship between the motivational level measured at the EEfRT and decision-making abilities at the IGT. Healthy participants were separated into two groups based on their ability to develop a strategy on the IGT. Recording neural activity during the EEfRT execution allowed for the definition of neural activity differences between subjects who developed a successful strategy at the IGT and subjects who did not.

### Relationship Between Decision-Making on the IGT and Behavioral Performances on the EEfRT

Behavioral analyses demonstrated that all participants, independently of their attribution groups, chose a mixture of HC/HR trials and LC/LR trials on the EEfRT. There was no difference between groups in the percentage of trials successfully completed, confirming that all participants were able to complete both the hard and easy tasks throughout the experiment. Therefore, the calibration of the number of presses did not negatively affect performance. Furthermore, there were no differences between favorable and undecided groups in the propensity to choose HC/HR or LC/LR trials. However, the pursuits of the analysis on the whole group showed a negative correlation between choosing HC/HR trials in the high probability condition to received gain (90%) and the netscore on the IGT. In other words, the more subjects make difficult choice in the high probability condition, the less likely they are to perform well on the IGT. Such a result suggests that the usual method of assigning participants into two or more groups according to their performance at the IGT may be somewhat artificial, and that decision-making performance has to be analyzed as a continuum to understand the underlying processes.

The likelihood to choose HC/HR in the high probability condition has been previously associated with anticipatory pleasure (Yang et al., 2014). Appetitive pleasure was positively correlated with the likelihood to make hard choices in high probability conditions, however, this study was conducted on subjects with subsyndromal depression (Yang et al., 2014). The current participants declared having no previous medical history of psychiatric disorders, substance abuse, alcohol abuse, neurological diseases, traumatic brain injury or stroke, and did not take any medication. As the pleasure anticipation was not controlled for in the current study with an appropriate scale, we cannot affirm its role in decision-making. However, we pose a plausible hypothesis that more poor performance on the IGT was associated with a stronger pleasure anticipation and a stronger reward sensibility. In IGT, the emotional processing in addition to the cognitive processing allows the development of the optimal strategy (Bechara et al., 2005; Buelow and Suhr, 2009). To succeed, subjects must learn that two decks are advantageous with small reward and small punishments and that two other decks are disadvantageous with larger immediate reward but a larger long-term punishments (Bechara et al., 1994). Deck composition drives the hypothesis that a disadvantageous strategy was the consequence of reward hypersensitivity. To respond to this, Bechara et al. (2000b) developed variants of the IGT. The original IGT is structured on the reward distribution, whereas the variant is structured on the punishment distribution. Indeed, the magnitude and frequency of immediate reward and punishment, according to several authors, confound long-term decision making (Singh and Khan, 2012). Therefore, the variant of the IGT appears to affect different performances, with more subjects having an impaired decision-making on the reward variant compared to the punishment variant (Bechara and Damasio, 2002). In concurrence with our findings, immediate reward seem to generate greater difficulty in long-term decision-making ability. These data confirm our behavioral hypothesis that decision-making alteration is generated by a stronger reward anticipation and a stronger reward sensitivity.

### Neural Mechanisms of Motivation

The ERP analysis was conducted to evaluate our hypothesis that motivation could influence reward sensitivity, which would result in poor decision-making abilities. Neural activity analyses were made on several ERPs, with the aim to describe and identify all elements that influence performance.

#### The P300 as a Neural Marker of Motivation

The P300 analysis during the EEfRT provides important elements for comprehension. First, the whole group analysis showed a result processing with a significant difference between the gain receptions and not receiving a gain. The gain reception induced a greater positive reaction compared to the absence of reception (Rigoni et al., 2010; Bland and Schaefer, 2011; Cui et al., 2013; Ferdinand and Kray, 2013; Mapelli et al., 2014). However, when this analysis was conducted on the groups, its presence appeared only on the undecided group. Indeed, surprisingly, only participants of the undecided group showed late outcome processing, with a greater sensitivity to the result. This observation is confirmed by the whole group analysis, which showed a significant negative correlation between P300 amplitude to the gain recording during the EEfRT and the net score on the IGT. However, we have previously demonstrated that P300 amplitude is proportional to the motivational level (Yeung and Sanfey, 2004). Per our hypothesis, it seems contradictory that the undecided subjects have the stronger motivational level and that its level is associated with poor decision-making abilities. However, when we reconsider this result by the prism of the various motivational concepts, this correlation appears more coherent. Indeed, the P300 amplitude represents a motivational state induced by the desire to obtain an immediate reward. Therefore, a greater sensitivity to the reward is translated by a greater P300 amplitude. The current participants exhibited a greater sensitivity to exogenous factors, causing a stronger extrinsic motivation (Ryan and Deci, 2000). The concepts of extrinsic and intrinsic motivation, proposed by Ryan and Deci (2000) and Ryan and Deci (2000), serve to distinguish between the interest originating from the activity itself and the interest caused by exogenous factors, two aspects of motivation that influence each other (Robinson et al., 2012). The entanglement between intrinsic and extrinsic motivation is still debated. However, exogenous factors could reduce the intrinsic motivation for the activity and could therefore have a negative impact on performance. It is plausible that the monetary incitation could negatively affect intrinsic motivation (Studer and Knecht, 2016). As a consequence, during IGT realization, participants with a stronger extrinsic motivation would favor more the decks with immediate gratifications, to the detriment of the future losses.

The P300 analysis during the IGT corroborates these observations. Indeed, P300 amplitude appears to be more important in the undecided group. Similarly, the P300 amplitude during the IGT realization is negatively correlated to the net score. This information could be reconciled with reward hypersensitivity and confirms our hypothesis on the behavioral analysis. However, if the P300 is sensitive to the outcome valence and amplitude, it is also a carrier of more complex cognitive information. Indeed, its amplitude is modified by the attention that the participant lends to the stimulus (Polich, 2007), without contradiction to its motivational aspect. The motivation increases, in a significant way, the interest for the stimulus with major consequences on attentional level. Therefore, based on these observations, it appears that the inability to develop an optimal strategy is associated with greater attention on the immediate outcome. Impacts on memory provides additional information to better comprehension, because the P300 is also assigned to the working memory updating after an unexpected event (Polich, 2004, 2007). In conclusion, the P300 reflects the attentional allocation process and the process of updating memory (Scharinger et al., 2017). Frequently, performance differences on the IGT could be explained by differences in cognitive abilities. More precisely, it was recognized that working memory played an important role on IGT performances (Bechara et al., 1998; Maia and McClelland, 2004). However, these observations were made on clinical populations, which could explain the discordance with our results. The updating of the working memory observed through the P300 was correlated with poor performance on the IGT. Decision-making ability seems to be influenced by the ability to filter the irrelevant distractors, rather than the ability to store immediate outcomes from decision-making in the working memory (Schmicker et al., 2017). This therefore explains why participants who developed an optimal strategy showed a blunted P300. This blunted P300 translates to less attention being paid to the immediate reward with a greater ability to filter distractors, in favor to an efficient long-term strategy.

The undecided group seemed to evaluate outcomes as more unexpected than the favorable group. As a consequence, the more they are sensitive to and surprised by the outcome, the more they take their decision as a function of the immediate outcome. This led to poor decision-making abilities with the immediate reward choice and negative consequences in the long term. This is in concurrence with a previous study, which showed a reward hypersensitivity induced inability to develop an optimal strategy (Bechara et al., 2002).

In the last step, source localization visually confirmed that one of the generators of the P300 wave was located in the ventromedial prefrontal cortex (vmPFC) (Horovitz et al., 2002; Polich, 2007; Wang et al., 2014). Indeed, comparison of vmPFC activity between groups showed a stronger activity during gain processing in the undecided group. This activity may reflect a processing in favor of the most appealing result (Rogers et al., 2004) in the undecided group. This confirms a reward sensitivity in the undecided group and that their behaviors are motivated by the reward perspective at the expense of punishments, compared to the favorable group.

#### Other ERPs Involved During the EEfRT

The exploratory analysis of the SPN during the EEfRT showed that, at the whole group level, the SPN was more negative for difficult compared to easy choices. This result suggests that difficult choices were linked to greater reward and, when participant made the choice of the difficulty, they were more hopeful to obtain the desirable outcome. This result is in line with a previous study in which the possibility to receive desirable outcomes induced greater anticipatory negativity (Fuentemilla et al., 2013).

Further exploratory analyses did not show any difference between groups. The lack of differences in the SPN analysis suggests the same level of commitment to the task between groups. However, the number of trials retained was too weak and did not allow for the processing of this information in any manner other than as an exploratory result. Indeed, we had to reject many trials during the ERP analysis due to artifacts induced by movement. It appears that following the task, subjects experience difficulties with being unable to move or blink.

Finally, we confirmed the presence of the FRN, with greater amplitude with a loss compared to a gain. The FRN did not differ between the groups, nor was its amplitude associated with the proportion of difficult choices. This result is in agreement with the previous literature, which described the FRN in the early outcomes processing (Gehring and Willoughby, 2002; Yeung and Sanfey, 2004; Holroyd et al., 2006). Furthermore, the FRN is indifferently observed during the IGT and EEfRT. Cumulatively, this data provides information on the unique role of the FRN in the outcome processing.

## Conclusion

Although we did not find that motivation directly influence decision-making performance at the IGT, behavioral and neuronal data provide evidence of a relationship between the propensity to focus only on the immediate outcomes and the development of an inefficient strategy on the IGT. Whether altered decision-making is a cause or a consequence of focusing on immediate outcomes remains to be explored. It is plausible that the behavioral differences on the EEfRT there were not significant in the current, healthy population could be observed in clinical population with important variation in their motivational level. Therefore, behavioral differences could provide categorical information, while ERPs bring a more dimensional approach, with a continuum between good and impaired decision-making abilities, as demonstrated by the correlation. The current investigation should be extended to a clinical population in order to verify this hypothesis.

## Data Availability Statement

The datasets generated for this study are available on request to the corresponding author.

## Ethics Statement

The studies involving human participants were reviewed and approved by Ethics Committee of Besançon University Hospital [authorized by the General Health Administration (ANSM 2016-A00870-51)]. The patients/participants provided their written informed consent to participate in this study.

## Author Contributions

JG, DG, MN, LP, PV, and EH conceived of and designed the experiments. JG, TC, and DG performed the experiments. JG, DG, NG, and TC analyzed the data. JG, DG, DB, JT, MN, CM, BT, MN, and EH contributed to writing of the manuscript.

## Conflict of Interest

The authors declare that the research was conducted in the absence of any commercial or financial relationships that could be construed as a potential conflict of interest.

## Footnotes

1 http://brainmapping.unige.ch/Cartool.php
